# EvtSNN: Event-driven SNN simulator optimized by population and pre-filtering

**DOI:** 10.3389/fnins.2022.944262

**Published:** 2022-09-29

**Authors:** Lingfei Mo, Zhihan Tao

**Affiliations:** FutureX Lab, School of Instrument Science and Engineering, Southeast University, Nanjing, China

**Keywords:** spiking neural network (SNN), event-driven, acceleration, simulator, unsupervised learning

## Abstract

Recently, spiking neural networks (SNNs) have been widely studied by researchers due to their biological interpretability and potential application of low power consumption. However, the traditional clock-driven simulators have the problem that the accuracy is limited by the time-step and the lateral inhibition failure. To address this issue, we introduce EvtSNN (Event SNN), a faster SNN event-driven simulator inspired by EDHA (Event-Driven High Accuracy). Two innovations are proposed to accelerate the calculation of event-driven neurons. Firstly, the intermediate results can be reused in population computing without repeated calculations. Secondly, unnecessary peak calculations will be skipped according to a condition. In the MNIST classification task, EvtSNN took 56 s to complete one epoch of unsupervised training and achieved 89.56% accuracy, while EDHA takes 642 s. In the benchmark experiments, the simulation speed of EvtSNN is 2.9–14.0 times that of EDHA under different network scales.

## 1. Introduction

Spiking neural networks (SNNs) (Maass, 1997) have attracted increasing attention because of their characteristics, including preferable biological interpretability and low-power processing potential (Akopyan et al., 2015; Shen et al., 2016; Davies et al., 2018; Moradi et al., 2018; Pei et al., 2019; Li et al., 2021; Pham et al., 2021). Compared to traditional artificial neural networks (ANNs), SNNs increase the time dimension so that they naturally support information processing in the temporal domain. To introduce the extra time dimension into the calculation, two methods are usually adopted: clock-driven and event-driven. The idea of clock-driven is to discretize the time and update the state of all neurons in each timestamp. The clock-driven method is widely used in the existing SNN frameworks (simulators) (Goodman and Brette, 2008; Hazan et al., 2018; Stimberg et al., 2019) because it is simulated by the iterative method which can be compatible with the differential equations of most neuron models. However, this method has two problems that cannot be ignored. Firstly, there is a conflict between simulation accuracy and calculation speed. The smaller the time step, the higher the simulation accuracy and the larger the calculation amount. Secondly, lateral inhibition cannot be effective on other neurons that fire lately in the same time slice.

In the event-driven method, the state of neurons is updated when spikes are received, which means that the sparsity of spikes can be fully utilized to reduce computations. The realization of event-driven simulation on hardware (Davies et al., 2018; Li et al., 2021) has the ability of parallel computing and the potential of low-power processing, but it is costly and less flexible than software. Our team previously proposed an event-driven software simulation framework EDHA (Event-Driven High Accuracy), whose core task is to maintain the pulse priority queue (Mo et al., 2021). During the simulation, the earliest spike is popped from the queue, and then postsynaptic neurons are updated independently. However, the high complexity of its single update limits the overall simulation speed.

In this paper, an event-driven software simulator named EvtSNN (Event SNN) is introduced, which includes two contributions. To begin with, neurons are clustered into populations, which means that intermediate results can be reused. In addition, pre-filtering is adopted to avoid unnecessary calculations according to the condition. After rewriting the framework code with the C++ programming language and combining these two innovations, the simulation speed of EvtSNN has been greatly improved. In the ablation experiment task, the processing capacity of EvtSNN(C++) reached 117.8 M spikes × fan-outs/s, which was 13 times that of EDHA(java). In the unsupervised training task of MNIST, the network (784–400) took 56 s to train one epoch with an accuracy of 89.59%, which is 11.4 times faster than EDHA.

In Section 2, we describe the related work, including unsupervised learning and supervised learning, as well as clock-driven and event-driven simulation. In Section 3, the principle of EDHA is reviewed, and two innovations are proposed to accelerate the event-driven simulation. Section 4 contains several comparison experiments and results. And the discussion is in Section 5. Finally, Section 6 summarizes the current work.

## 2. Related works

The learning methods of SNN mainly include supervised learning and unsupervised learning. Supervised learning is similar to the traditional ANN, which can be trained by the gradient back-propagation (BP) method. However, the spike is non-differentiable, so ANN to SNN (Sengupta et al., 2019; Deng and Gu, 2021) or surrogate gradient (Neftci et al., 2019) is often used to handle this problem. Unsupervised learning refers to the learning style of neurons in biology, which has better biological interpretability. Similar to EDHA, EvtSNN pays more attention to biological interpretability, and currently mainly supports unsupervised learning rules, such as the spike time-dependent plasticity (STDP) (Masquelier and Kheradpisheh, 2018) learning rule.

Most SNN simulators need to deal with the temporal dimension. Clock-driven and event-driven approaches are often used to deal with this problem. Owing to high model compatibility, the clock-driven method is mostly used in software SNN simulation frameworks, such as Brian (Goodman and Brette, 2008), Brian2 (Stimberg et al., 2019), and BindsNET (Hazan et al., 2018), etc. Brian and Brian2 were frameworks based on code generation, which can specify the neuron dynamic equation and synapse update rule to generate corresponding codes. On the other hand, BindsNET was based on PyTorch (Paszke et al., 2019) and implemented the behaviors of neurons and synapses by writing code. Its advantage is that Graphics Processing Unit (GPU) acceleration can be conveniently carried out based on PyTorch. Although with high model compatibility, the clock-driven method also has some problems. Firstly, the existence of time-slice limits the simulation accuracy, and the calculations increase inversely with the decrease of time-step. Secondly, when using lateral inhibition, multiple neurons in the same layer can activate in a time step, leading to multiple winning neurons that are unfavorable for training.

The event-driven method is mostly used in Field Programmable Gate Array (FPGA) and Application Specific Integrated Circuit (ASIC). FPGA is an expensive and flexible chip with limited resources, which is suited for laboratory prototype validation and not for actual deployment (Pham et al., 2021). Some researchers have made ASIC for simulating SNN, such as DYNAPs (Moradi et al., 2018), TrueNorth (Akopyan et al., 2015), and Loihi (Davies et al., 2018), etc. The power consumption of these is at the milliwatt level, but they have expensive costs for design, and it is not convenient to add new functions.

Event-driven simulation can take advantage of the sparsity of spike events and neural connections. EDHA is an event-driven framework proposed by our team earlier, whose core task is to maintain the spike priority queue. Without the concept of time-slice, it solves the problems of lateral suppression failure and the conflict between accuracy and speed in the clock-driven method. However, there are large calculations in the update of neurons in EDHA, which limits the overall simulation speed. Therefore, two innovations have been proposed to solve this problem.

## 3. Methods

### 3.1. Workflow of EDHA framework

The SNN simulation framework needs to deal with additional time dimensions. The clock-driven method is similar to polling, while the event-driven method calculates only when pulse events are received.

**Figure 1:**
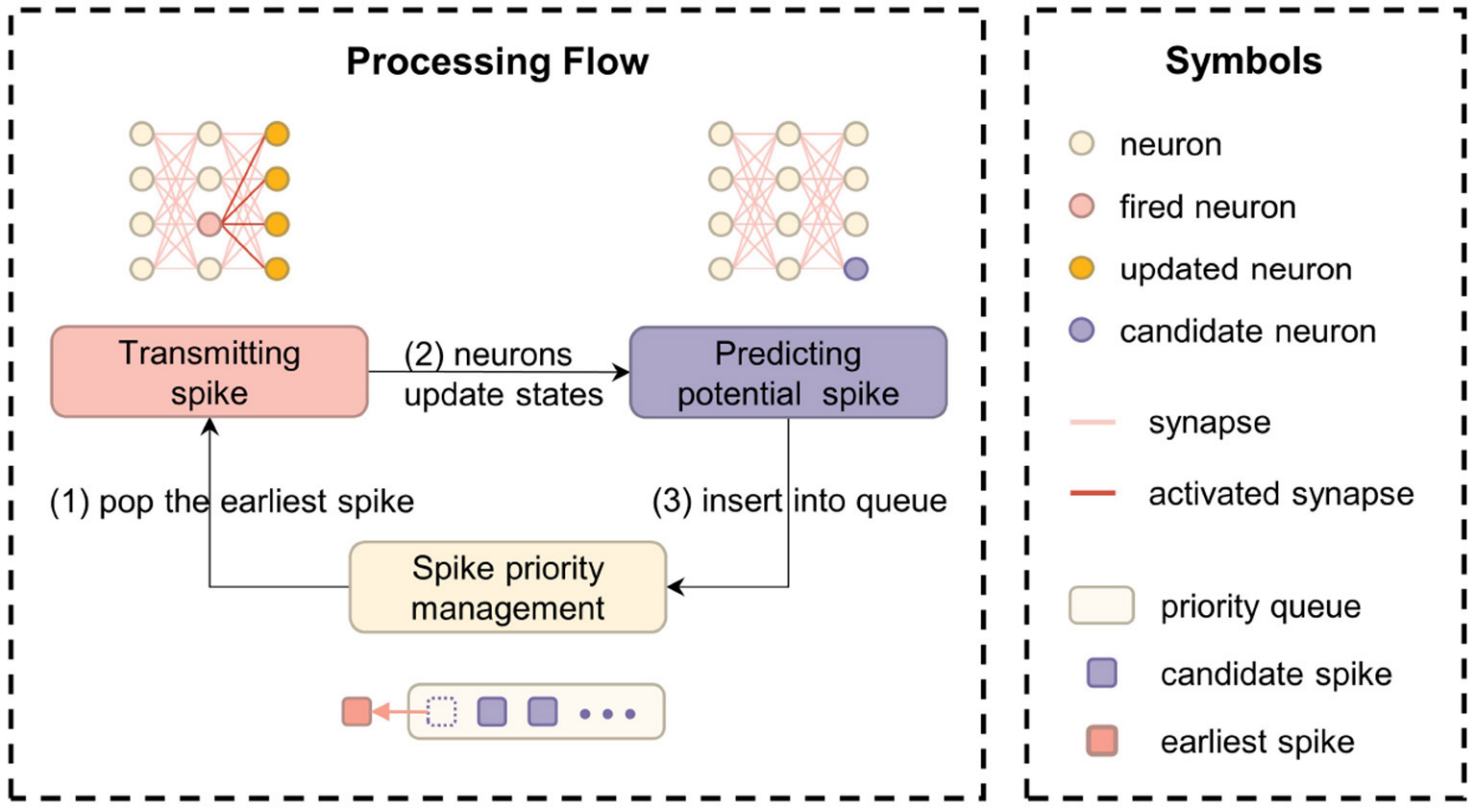
The computational flow chart of EDHA and EvtSNN, which core task is to maintain the spike priority queue.

As an event-driven simulation framework, the core task of EDHA is to maintain the spike priority queue. The specific steps are as follows: (1) take out the earliest spike event that should be issued from the priority queue; (2) update the neurons which are connected behind the fired neuron (i.e., post-synaptic neurons); (3) adjust the priority queue elements according to the predicted spike information. Figure 1 is a flow chart of the above processing details.

### 3.2. Neuron and synapse model

The leaky integrate-and-fire (LIF) model (Gerstner et al., 2014; Mo et al., 2021) was adopted in this paper, which is represented by Equations (1) and (2). δ(*t*) in the formula is the impulse function, and other parameters are explained in Table 1. It should be noted that there is no dependence on membrane potential in Equation (2), which facilitates the derivation of other formulas.


(1) $$\begin{array}{l} \{\begin{array}{ll} \frac{dv}{dt}=-\frac{v}{\tau_{v}}+g_{E} & v<v_{th}\\ v=v_{reset} & v\geq v_{th} \end{array} \end{array}$$

(2) $$\begin{array}{l} \{\begin{array}{ll} \frac{dg_{E}}{dt}=-\frac{g_{E}}{\tau_{g}}+C\sum_{i,j}w_{i}\delta (t-t_{i,j}) & v<v_{th}\\ g_{E}=0 & v\geq v_{th} \end{array} \end{array}$$

**Table 1:** Explanation and experimental value of the parameters in the LIF neuron model.

**Parameter**	**Explanation**	**Section 4.1**	**Section 4.2**	**Section 4.3**
*v*	Membrane potential			
*g*_*E*_	Excitatory conductance			
τ_*v*_	The leaky time constant of voltage	20	20	50
τ_*g*_	The leaky time constant of conductance	1	5	5
*C*	Conductivity gain coefficient	1	1,000/(*N·Fr*)	Variable
*w*_*i*_	Weight connect to presynaptic neuron *i*	Rand (0, 0.3)	1	Rand (0.45, 0.55)
*t*_*i,j*_	The time of jth spike of presynaptic neuron *i*			
*v*_*th*_	Activation threshold of neuron			
*v*_*reset*_	Reset potential after fire	0	0	0
θ_0_	Adaptive threshold baseline	40	100	75
θ	Increment of adaptive threshold	0.5	0.5	2.5
τ_θ_	Time constant of adaptive threshold	1e7	1e7	1e7

The adaptive threshold method is often used to avoid a few neurons firing too frequently. It is increased by the fixed item (θ) at each fire, and otherwise exponentially decays toward θ_0_ with a time constant (τ_θ_). It is more difficult to generate new spikes after increasing the threshold, which is beneficial for other neurons' learning (Masquelier and Kheradpisheh, 2018). Owing to large τ_θ_, the decay of *v*_*th*_ in a short time can be ignored in practice, which simplifies some calculations.

The plasticity synaptic model is an important part of unsupervised learning. Like EDHA, the STDP learning rule was employed in this paper, see formula (3) (Mo et al., 2021). In which σ_+_ and σ_−_ are the amplitude constants of LTP and LTD, τ_+_ and τ_−_ are the time constant, and *t*_*pre*_ and *t*_*post*_ are the firing time of the pre-/post-synaptic neuron.


(3) $$\begin{array}{l} \Delta w=\{\begin{array}{ll} \sigma_{+}\cdot e^{-\frac{t_{post}-t_{pre}}{\tau_{+}}} & t_{pre}<t_{post}\\ \sigma_{-}\cdot e^{-\frac{t_{pre}-t_{post}}{\tau_{-}}} & t_{pre}>t_{post} \end{array} \end{array}$$

### 3.3. Update steps of neuron

**Figure 2:**
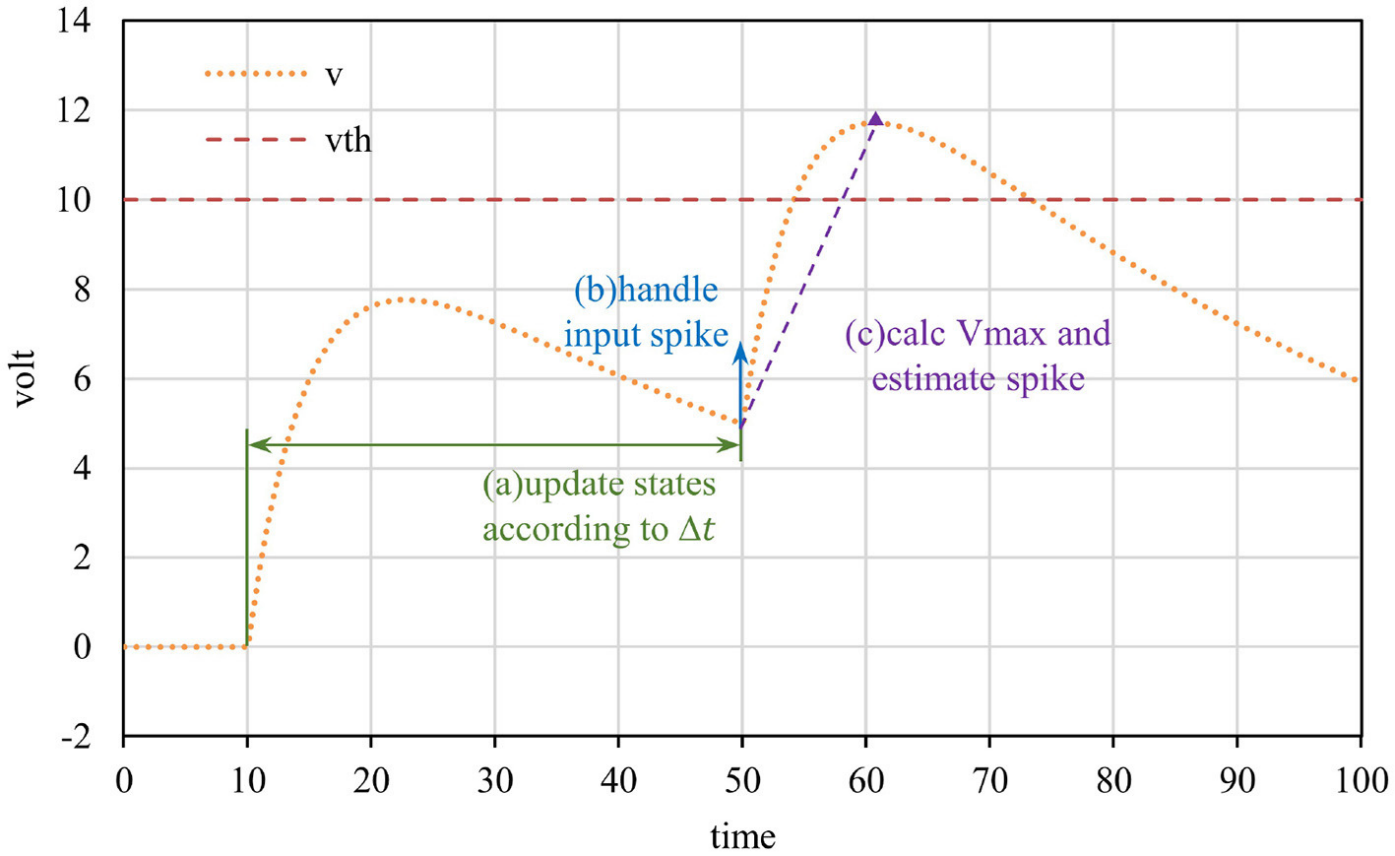
Event-driven neuron updated diagram. The neuron is updated when a spike is received, which consists of three sub-steps: updating states, processing the input spike, and predicting the potential spike.

In step (2) of the event-driven framework workflow, there are three sub-steps: (a) calculate the current state according to the update interval, (b) handle the currently input spike event, and (c) estimate the potential pulse based on the current state. Figure 2 shows the diagram of the above three sub-steps, and the formula of each step is related to the neuron model. It is important to note that the neuron's membrane potential may continue to increase for some time after receiving a single spike, so sub-step (c) is required to estimate the potential pulse of neurons.

Unless received spike or activated, *v* and *g*_*E*_ will not change instantaneously, and Equations (4) and (5) are obtained. *T* represents the time of the currently received spike. At any time from *T* to the next pulse received, the state can be solved by the above two equations, which is the computing formula in sub-step (a).


(4) $$\begin{array}{l} \begin{array}{c} v\left(T+\Delta t\right)=v\left(T\right)\cdot e^{-\frac{\Delta t}{\tau_{v}}}+g_{E}\left(T\right)\cdot \frac{\tau_{g}\tau_{v}}{\tau_{g}-\tau_{v}}\left(e^{-\frac{\Delta t}{\tau_{g}}}-e^{-\frac{\Delta t}{\tau_{v}}}\right) \end{array} \end{array}$$

(5) $$\begin{array}{l} g_{E}\left(T+\Delta t\right)=g_{E}\left(T\right)\cdot e^{-\frac{\Delta t}{\tau_{g}}} \end{array}$$

In sub-step (b), the influence of the pulse on the current state is calculated. According to Equation (1) and (2), *v* is unchanged and *g*_*E*_ increases by *Cw*_*i,j*_ instantaneously when received spike.

In sub-step (c), the neuron needs to estimate whether it can generate a new spike according to current states. After receiving the spike, the conductance of the neuron increases, which indirectly promotes the increase of the membrane potential, and it is difficult to predict the precise timing of the pulse firing. Instead, the method of calculating the peak value of voltage was adopted. If the peak exceeds the threshold, combined Equation (4), the dichotomy method was employed to calculate the exact spike time (Mo et al., 2021).

Here are the details of solving peak membrane potential. Equation (1) shows that the membrane potential is affected by the self-attenuation term and conductance. The voltage will increase when the conductance is large and plays a leading role. In the case no subsequent spikes are received, the conductance *g*_*E*_ decays exponentially to 0. In other words, if the conductance is large enough, the voltage will rise first and then fall, and reach the peak during this period. In other cases, potential decreases monotonically under the influence of attenuation term. According to Equation (4), the extreme point *t*′ and the corresponding peak membrane potential were obtained, i.e., Equations (6) and (7).


(6) $$\begin{array}{l} t^{{\;}^{\prime }}=\frac{\tau_{g}\tau_{v}}{\tau_{g}-\tau_{v}}\cdot \left[\ln\left(\frac{\tau_{g}}{\tau_{v}}\right)+\ln\left(1-\frac{\tau_{g}-\tau_{v}}{\tau_{g}\tau_{v}}\cdot \frac{v\left(T\right)}{g_{E}\left(T\right)}\right)\right] \end{array}$$

(7) $$\begin{array}{l} v_{max}=\{\begin{array}{ll} g_{E}(T)\tau_{v}{\left[\frac{\tau_{g}}{\tau_{v}}(1-\frac{\tau_{g}-\tau_{v}}{\tau_{v}\tau_{g}}\cdot \frac{v(T)}{g_{E}(T)})\right]}^{-\frac{\tau_{v}}{\tau_{g}-\tau_{v}}} & t^{\prime }>0\;\\ v(T) & \text{else} \end{array} \end{array}$$

In the above formula, there is a large computational complexity in sub-step (a) and (c), which is the bottleneck in the whole simulate computation. However, it is found that two points can be optimized in the actual calculation. First, there are many same Δ*t* of postsynaptic neurons (fan-outs), which means the decay factors can be reused. Second, the complicated calculation in sub-step (c) can be omitted if the neuron will not be activated in the current state. Therefore, in the following sections, the two innovations of population computing and pre-filtering were proposed, which avoid repeated calculation and unnecessary calculation, respectively.

### 3.4. Population computing

The supported connection style in EDHA was the fine-grained (one-to-one) connection between neurons, and it is extremely flexible. However, the coarse-grained (layer-to-layer) connection is usually adopted due to its convenience. Furthermore, all neurons in the population will be updated when they receive a spike so that they have the same update interval. According to Equations (4) and (5), the decay factors of neurons in the population can be reused due to the same update interval.

To cooperate with the population, the local priority queue was introduced, which sorted the pulses in the population and provided the earliest spike to the global queue. It slightly increases the complexity of the program and reduces some flexibility. However, using the concept of the population to manage multiple neurons achieves high cohesion and low coupling, which is beneficial to follow-up work.

In addition, due to the introduction of the concept of population, the simulation could support more operations, such as the delayed update of the adaptive threshold. In EDHA, neurons update the threshold when receiving a spike, but usually, the threshold change is very small. The delay update threshold has little effect on the simulation accuracy while avoiding a lot of exponential calculations.

### 3.5. Pre-filtering

In sub-step (c), the peak potential is calculated according to the current state. In most cases, the peak potential of the neuron will not exceed the threshold after receiving the spike, which means that many peak calculations are unnecessary. Therefore, the judgment condition of pre-screening was proposed to filter out unnecessary calculations at a low cost.

At time *t*_*f*_, the membrane potential increases to *v*_*th*_, and inequality (8) can be derived. This inequality is a necessary condition for activation. If the current conductance does not meet this condition, it means that the neuron will not generate a spike in the future unless more input events are received.


(8) $$\begin{array}{l} v^{{\;}^{\prime }}\left(t_{f}\right)=-\frac{v\left(t_{f}\right)}{\tau_{v}}+g_{E}\left(t_{f}\right)\geq 0 \end{array}$$

Inequality (8) can be used as the condition of pre-filtering, but it does not involve other information. For example, when the difference between *v* and *v*_*th*_ is large, a greater *g*_*E*_ is required to boost *v*. When voltage attenuation is neglected, it is easy to get the contribution of conductance to the potential which is shown in inequation (9). After integration and arranging, inequality (10) is obtained, which reflects the contribution of conductance to membrane potential. Combining inequality (8), the judgment condition (11) can be obtained finally.


(9) $$\begin{array}{l} \frac{dv}{dt}=-\frac{v}{\tau_{v}}+g_{E}\leq g_{E} \end{array}$$

(10) $$\begin{array}{l} \Delta v\leq \int g_{E}dt=\int -\tau_{g}\frac{dg_{E}}{dt}dt=-\tau_{g}\Delta g_{E} \end{array}$$

(11) $$\begin{array}{l} g_{E}\left(T\right)\geq g_{E}\left(t_{f}\right)+\frac{v\left(t_{f}\right)-v\left(T\right)}{\tau_{g}}\geq \frac{v_{th}}{\tau_{v}}+\frac{v_{th}-v\left(T\right)}{\tau_{g}} \end{array}$$

**Figure 3:**
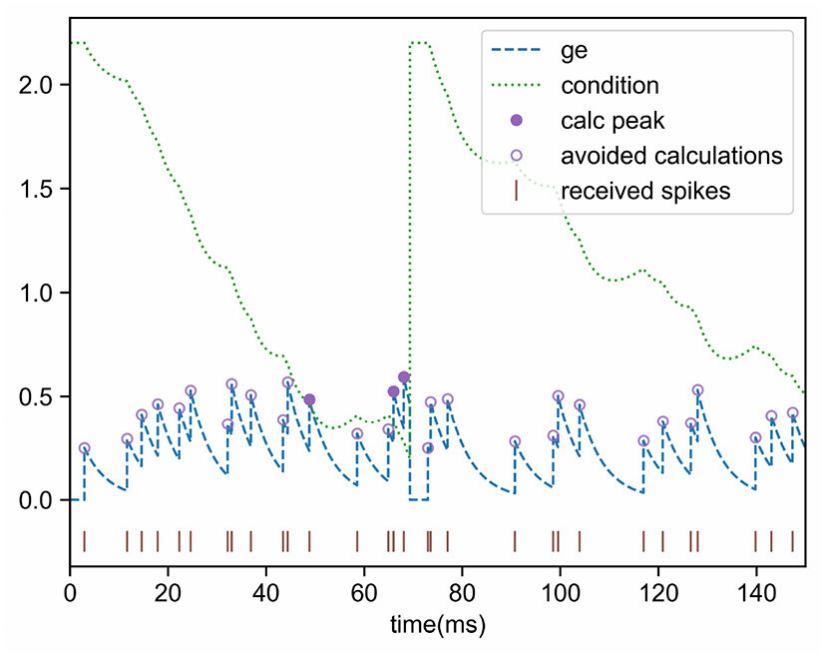
Effect of conductance pre-filtering. The peak value needs to be calculated only when the updated conductance is greater than the condition.

Inequation (11) is also an essential condition for the neuron to activate and then emit a spike. If the current state does not meet this condition, no pulse will be generated in the future (unless more input events are received), which means that complicated peak calculations can be ignored. This inequality makes use of most information, such as voltage, threshold, and two time-constants (the “leak” terms τ_*v*_ and τ_*g*_), and it is a good pre-filtering condition. Figure 3 shows the pre-filtering effect, and only a few cases (the purple solid dots) need to calculate the peak value, thus avoiding unnecessary computational overhead.

### 3.6. Method summary

**Figure 4:**
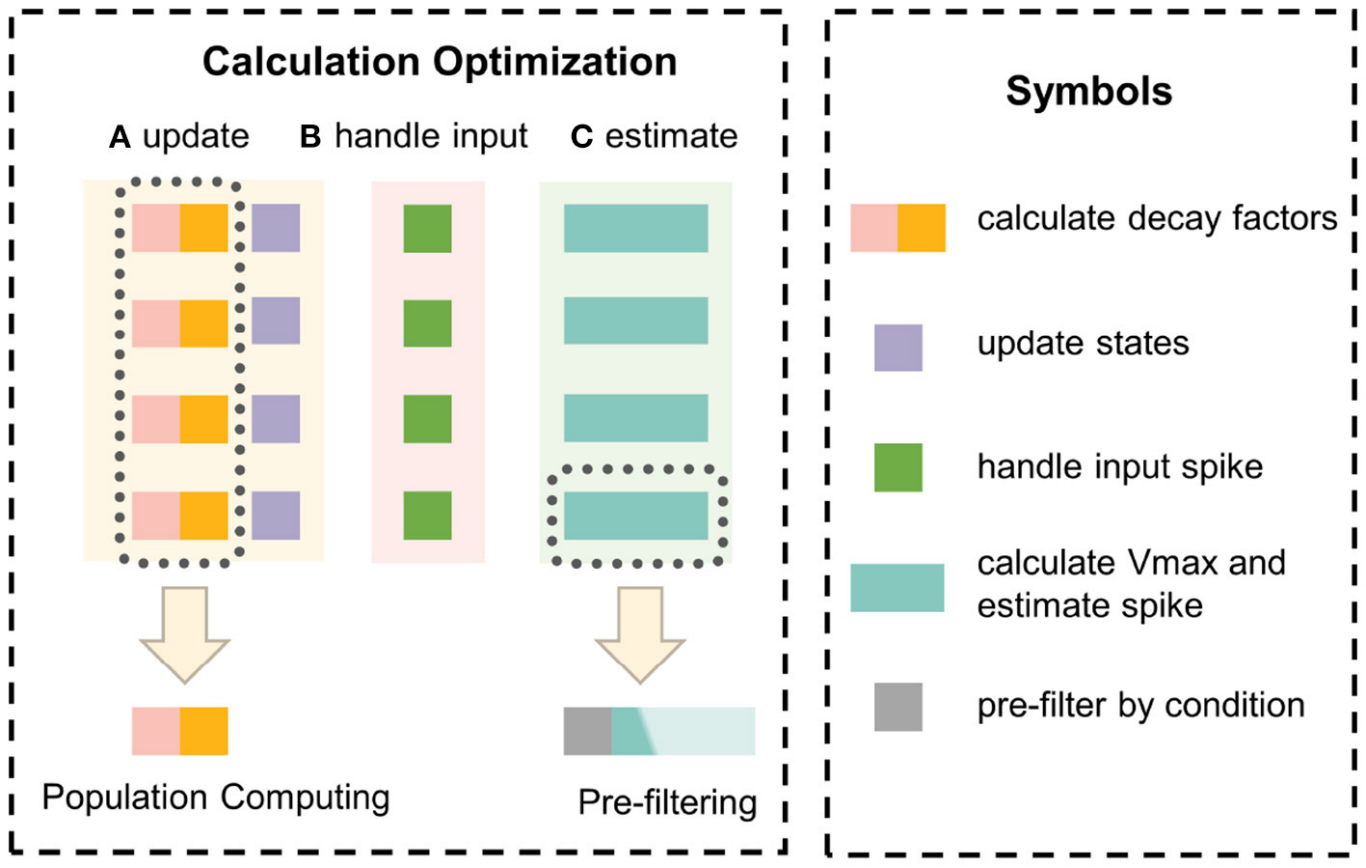
The optimization principle of innovation points. Population computing uses the hidden information of the same update interval to reuse decay factors to avoid repeated calculations. Pre-filtering adopts a low-cost judgment condition to filter out unnecessary calculations to reduce the average time-consuming.

In this section, the principle of the event-driven simulator EDHA is reviewed, and two innovations are proposed to optimize the problem of a large amount of calculation in a single update, which is shown in Figure 4. Based on EDHA and the above two optimizations, we propose the new simulation algorithm, named EvtSNN.

## 4. Experiments and results

In this section, Brian2 (python), BindsNET (python), and EDHA (java) were selected as comparison frameworks. To be fair, each framework was tested on the central processing unit (CPU) platform with one thread. The test platform is a workstation with Intel Xeon Silver 4215R@3.2 GHz CPU and the operating system is Ubuntu20.04. The parameters used in the experiments are shown in Table 1. The test programs of this section are shown in http://www.snnhub.com/FutureX-Lab/EvtSNN-exe.

In Brian2 simulator, one can specify different device as the backend, including runtime (cython or numpy), cpp_standalone, genn and so on. For the cpp_standalone and genn backends, they can avoid repeating compilation by setting *build_on_run* to False, but this approach cannot adjust the parameters of the next run according to the simulation results of this run. The runtime (cython) is the relatively fastest backend when the simulation time is short, and it is also selected as the default backend of Brian2 in the follow-up experiments. Furthermore, without changing the network structure and parameters, an empty function could be used to mask *before_run* after *run(0ms)*, which can avoid generating duplicate code, named Brian2*.

### 4.1. Performance test

The network structure used in the test included an input neuron layer (200 neurons), an output neuron layer (200 neurons), and fully connected synapses. With a 10 Hz average fire rate and 10,000 ms simulation time, there were 20 k spikes in total. For testing, we used the algorithm described in Bautembach et al. (2020) for generating the input spikes to the networks.

Table 2 shows the performance test results of the above four simulators. It can be seen that the simulation time of the clock-driven framework changes inversely with dt. When dt was reduced from 1.0 to 0.01 ms, the simulation accuracy was improved, but the simulation time was increased to nearly 100 times. Thanks to no time-step limitation, the event-driven simulator has extremely high simulation accuracy, which was almost the same as the output spikes of the clock-driven simulator with dt = 0.01 ms (~511). And the computation of event-driven simulator is only affected by the number of spikes and neuron fan-outs. According to Table 2, the processing capacity of EvtSNN reached 20 k×200/0.033958 s = 117.8 M spikes×fan-outs/second. Compared with EDHA, the performance of EvtSNN has greatly improved without loss of simulation accuracy.

**Table 2:** Performance test results of simulators.

**Simulator**	**Method**	**dt (ms)**	**Time-consuming (s)**	**Output spikes**
BindsNET	Clock-driven	1.0	2.353	499
		0.1	22.486	508
		0.01	231.169	510
BindsNET-GPU	Clock-driven	1.0	4.068	499
		0.1	38.851	508
		0.01	383.747	510
Brian2	Clock-driven	1.0	0.667	587
		0.1	3.169	519
		0.01	30.284	511
Brian2*	Clock-driven	1.0	0.312	587
		0.1	3.035	519
		0.01	29.915	511
Brian2GeNN	Clock-driven	1.0	19.630	587
		0.1	21.787	519
		0.01	49.443	511
EDHA	Event-driven	None	0.439	510
EvtSNN(ours)	Event-driven	None	0.034	511

In addition, we compare the results of Brian2 and BindsNET using GPU acceleration. Among them, Brian2GeNN (GPU) takes a long time to compile, and when dt is reduced from 1.0 to 0.01 ms the time-consuming increment of Brian2GeNN is similar to that of Brian2 (CPU), which means that the processing capacity of Brian2GeNN has not been significantly improved. At the same time, the speed of BindsNET-GPU is not as fast as that of BindsNET (CPU). It can be seen that in some cases, such as small networks and sparse pulses, using the GPU does not make the SNN simulation faster.

### 4.2. Benchmarking

The amount of computation of event-driven simulation is related to the number of spikes and neuron fan-outs. Theoretically, when the number of neurons in each layer increases and the firing rate of the input layer remains, the time-consuming increases in square trend. In this section, the simulation experiments of different scale networks were designed. The time step (dt) of Brian2 and BindsNET was set to 1.0 ms.

The structure of the network was two neuron layers, which were fully connected. We marked the number of neurons in both layers as *N* and the firing frequency of the input layer as *Fr*. Under different *N* and *Fr*, we recorded the execution time of simulating the network for 1,000 ms. To avoid spike number drastic change, the impact factor of weight (i.e., *C*) changed inversely according to *N* and *Fr*, thus stabilizing the firing frequency of the output layer.

**Figure 5:**
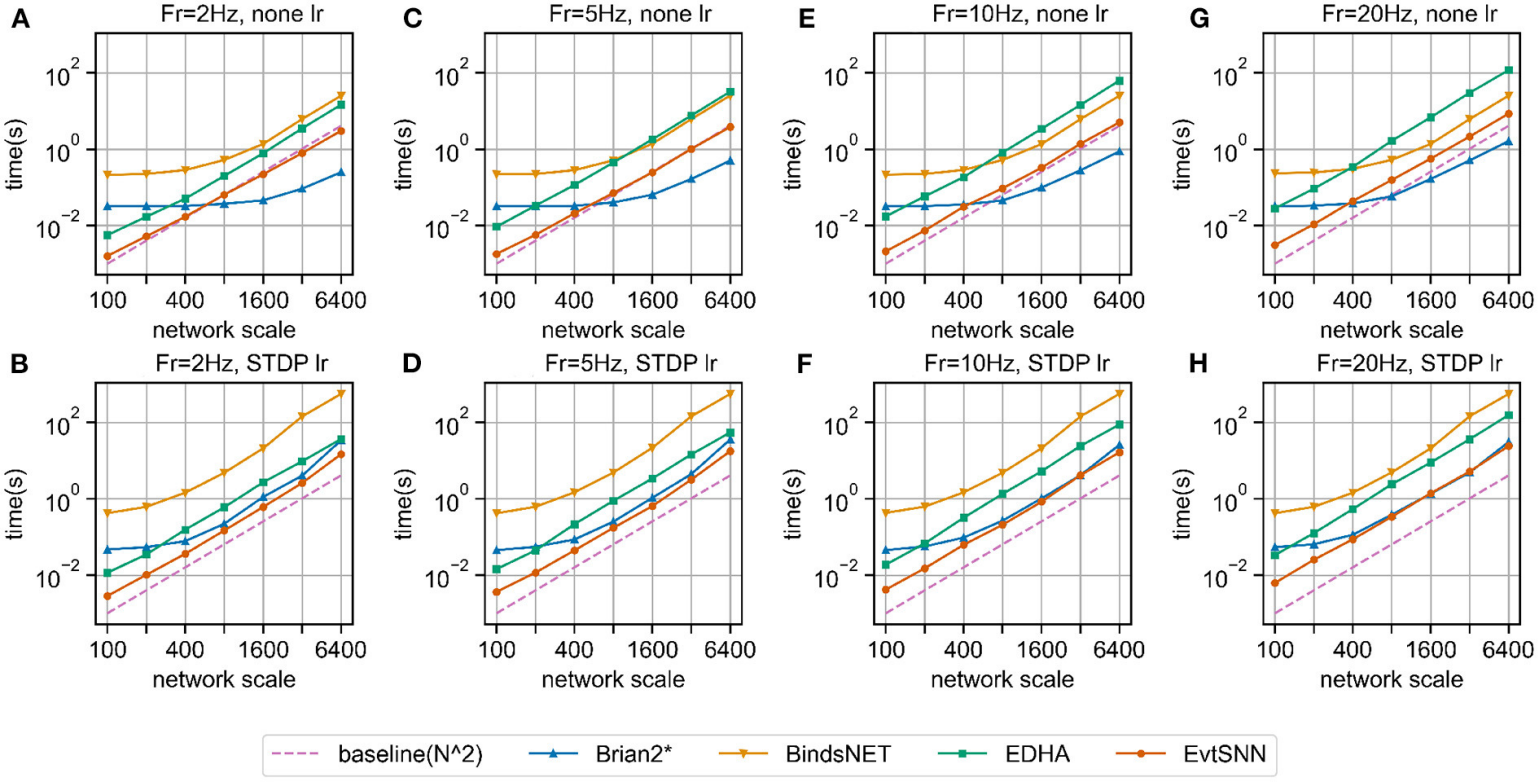
Benchmark simulation results. **(A–D)** No learning rule, the firing frequency of input layer neurons is 2, 5, 10, and 20 Hz, respectively. **(E–H)** Using the STDP learning rule, the firing frequency is 2, 5, 10, and 20 Hz, respectively.

The experimental results are shown in Figure 5. The horizontal and vertical coordinates of the chart were logarithmic coordinates, which could easily see the time-consuming growth trend. As predicted by the previous theory, the time consumption of the four simulators increased approximately square with the increase of the number of neurons (purple dotted baseline). Without the learning rule, in the case of small network scale (e.g., hundreds of neurons), the fastest simulator was EvtSNN, otherwise, it was Brian2*. When *Fr* increased (e.g., 20 Hz), compared with the clock-driven simulator, the performance of EvtSNN and EDHA decreased relatively. EvtSNN was the fastest framework at different *N* and *Fr* when using the STDP learning rule. The speed of EvtSNN is 2.9–14.0 times that of EDHA under the same calculation flow.

Brian2* performs better when it comes to large-scale network simulation, but the following points should be noted. Firstly, the statistical time taken by Brain2* does not include the time taken to generate the code, so it appears to be faster. In many cases it is necessary to change the run parameters, at which point Brian2 needs to recompile the code, so the extra time taken to compile cannot be ignored. Secondly, the input layer in the benchmark experiments had the same frequency of pulse delivery for each neuron, and there was no channel sparsity, so the event-driven framework, EvtSNN, did not take full advantage of sparse computation. Finally, the time step(dt) of the clock-driven framework in the benchmark experiments is 1.0 ms, and decreasing dt for higher simulation accuracy will increase the time consumption inversely; whereas EvtSNN has no time step limitation and has a fixed time consumption and high simulation accuracy.

### 4.3. Unsupervised training task on MNIST dataset

**Figure 6:**
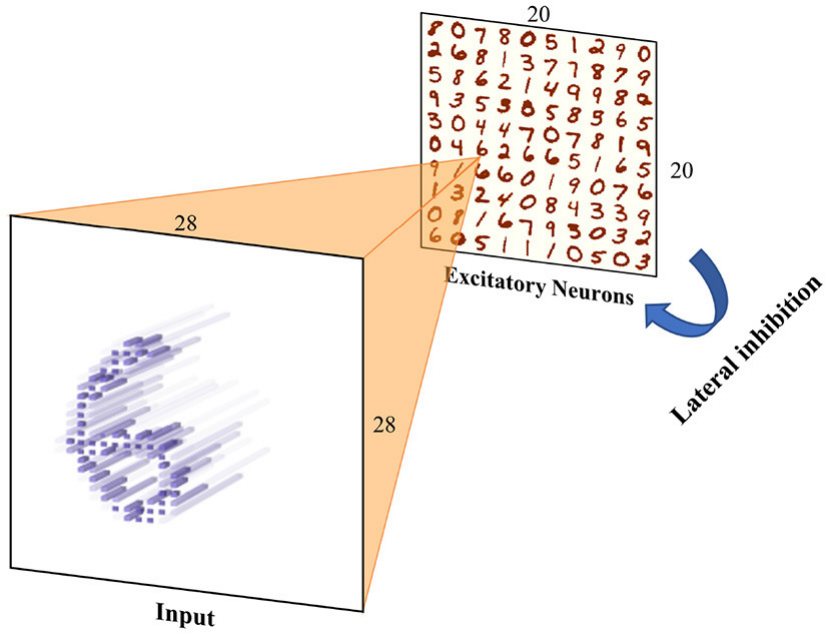
Unsupervised training network structure. Input images are encoded into spikes and then fed into the spiking neural network. The network includes an input layer and an output layer (excitation layer), there are synapses with learnable weights between them. Any neuron in the output layer has inhibitory connections with fixed weights to the others to achieve lateral inhibition.

In this section, the performances of several simulation frameworks were compared on the unsupervised training task of MNIST. The network mainly included the input neuron layer, output neuron layer, feature synapses layer, and suppression layer, which was inspired by Diehl's paper (Diehl and Cook, 2015). In EDHA and EvtSNN, the suppression layer was replaced by direct lateral inhibition, because it can be efficiently realized by the event-driven framework, and the network structure after modification is shown in Figure 6. The training code for Brian2 and BindsNET come from https://github.com/zxzhijia/Brian2STDPMNIST and https://github.com/BindsNET/bindsnet, respectively.

The MNIST dataset is image style data with 28 × 28 input pixels and 60,000 training samples (LeCun et al., 1998). Before being fed to the spike neural network, the image data should be encoded in spikes format. In EDHA, to reduce the number of input spikes and the amount of event-driven calculation, the time encoding method was adopted, and each pixel was coded into at most one pulse. The average pulse number of samples encoded in time encoding is far less than that of frequency coding, which can greatly reduce the calculation and speed up the simulation of the event-driven framework.

**Figure 7:**
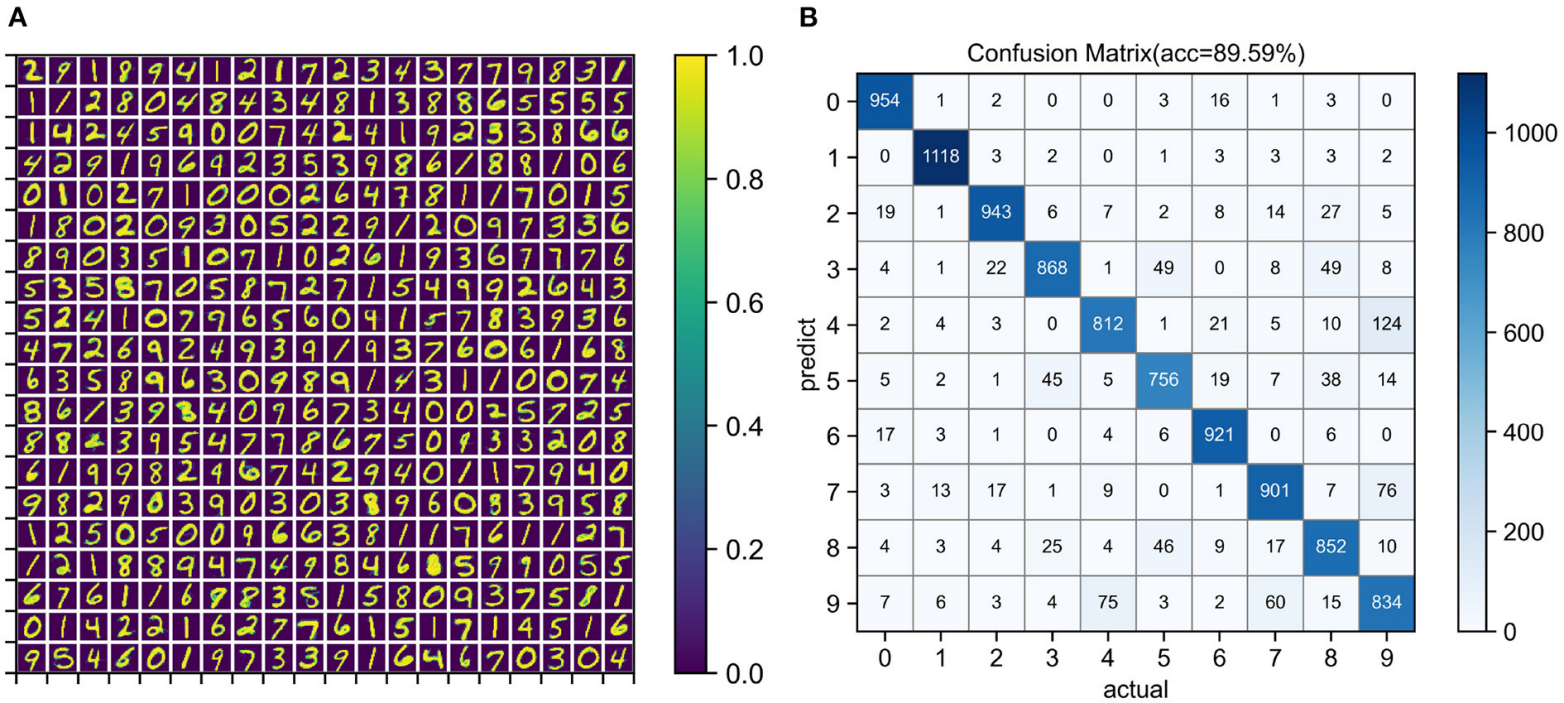
**(A)** Weight visualization after 1 epoch of unsupervised learning on the MNIST training set. **(B)** Confusion matrix for classification on the MNIST test set. The network size used for training and testing is 784–400.

Unsupervised training configuration, simulation time, and training results are shown in Table 3. The number of neurons in the input layer is the same as the number of image pixels, which is 784. The more neurons in the output layer, the better the expressive ability of the network. When the output layer has 400 neurons, the network training speed is fast and the classification accuracy is acceptable. After one epoch of training, the test accuracy of four simulators was similar. Training on the EvtSNN framework took only 56 s, which was much faster than other frameworks. In this task, EDHA and EvtSNN use the same parameters, the latter is 11.4 times faster (with time encoding) and 19.1 times faster (with frequency encoding) than the former. Figure 7A is the weight visualization after 1 epoch of training. It can be seen that the features of the image have been learned by the network and stored in the weights. Figure 7B is the confusion matrix on the test set, with an accuracy of 89.59%.

**Table 3:** Comparison of simulators performance in task of MNIST unsupervised training (1 epoch).

**Simulator**	**dt(ms)**	**Encode method**	**Sample duration (ms)**	**Average spikes**	**Accuracy (%)**	**Training time (s)**
Brian2	0.5	Frequency	350	2285.64	87.90	1.538E+05
Brian2*	0.5	Frequency	350	2285.64	87.90	1.479E+04
BindsNET	0.5	Frequency	250	1632.60	90.10	8.274E+04
BindsNET-GPU	0.5	Frequency	250	1632.60	88.58	3.288E+04
EDHA	None	Frequency	350	2285.64	89.71	9.128E+03
EDHA	None	Time	100	136.54	88.86	6.418E+02
EvtSNN(ours)	None	Frequency	350	2285.64	89.19	4.790E+02
EvtSNN(ours)	None	Time	100	136.54	89.59	5.637E+01

After turning on the GPU, with the support of PyTorch for GPU, the speed of BindsNET-GPU is 2.5 times faster than that of BindsNET. In this experiment, it is necessary to judge whether the network has pulse output after inputting the sample, so Brian2GeNN needs to compile and run to obtain the simulation results. However, each compilation takes tens of seconds, which is a considerable overhead, so no relevant experiments have been done.

**Figure 8:**
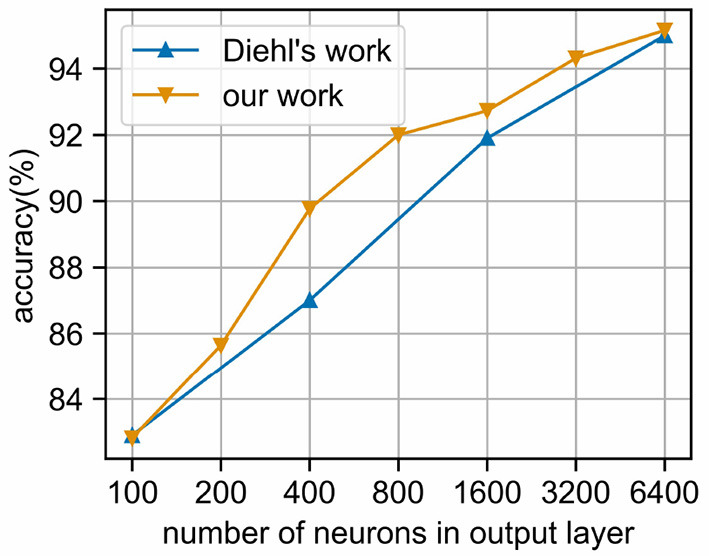
Classification accuracy of MNIST under different output layer scales. We use the EvtSNN framework combined with time coding for simulation, and the accuracy is no less than the Brian framework and frequency coding in Diehl's paper.

As shown in Figure 8, when the number of neurons in the output layer is increased, the classification accuracy of the network will be improved. Similar to the results of Diehl and Cook (2015), our network can achieve 95.16% accuracy when using 6,400 neurons, which demonstrates the feasibility of event-driven simulation combined with time coding.

## 5. Discussion

### 5.1. Simulation accuracy

**Figure 9:**
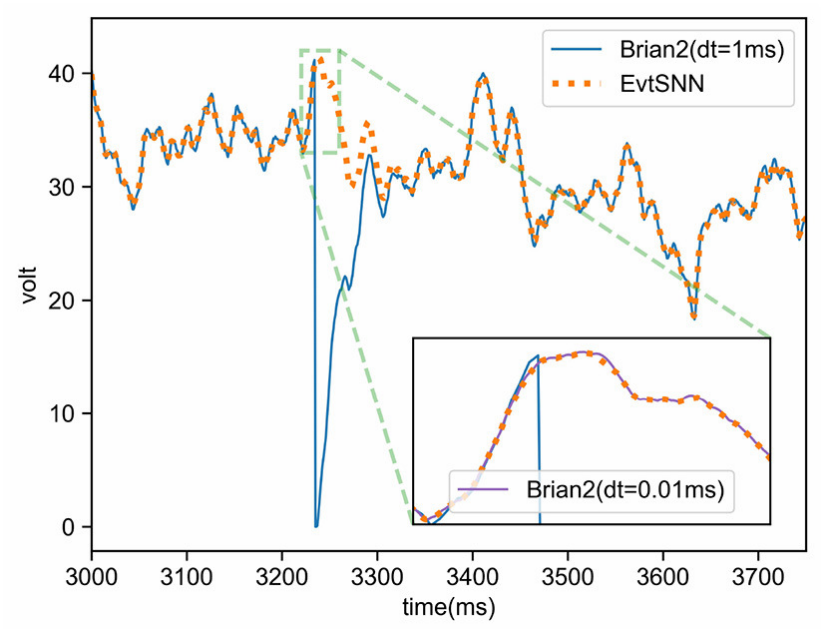
Voltage curves of neurons in Brian2 and EvtSNN simulations. The clock-driven framework Brian2 has higher simulation accuracy when using smaller time steps (e.g., dt = 0.01 ms), and its voltage is close to the result of the event-driven framework EvtSNN. When using a larger time step (e.g., dt = 1 ms), the simulation accuracy of Brian2 decreases and leads to changes in the firing pattern.

In the experiment of Section 4.1, the membrane potential of neurons during the simulation of Brian2 and EvtSNN were recorded and plotted, as shown in Figure 9. It can be seen that the voltage variation trend in the two simulations is basically the same, but sometimes there are small errors that affect spike delivery. Interestingly, even if the pulses do not match, the voltage will tend to be consistent after a while. This may be due to the decay of membrane potential. As time passes, the subsequent state is mainly affected by the input pattern rather than the initial state.

The simulation accuracy of the clock-driven method is limited by the time step, which may cause a small number of spike mismatches. However, in most cases, there are similar voltage curves in clock-driven (Brian2) and event-driven (EvtSNN) simulation, and the overall spike pattern is not much different, which means that the error of clock-driven simulation could be ignored many times. To sum up, there are some simulation errors in the clock-driven method, which can be ignored in most cases; the precision of the event-driven method can be very high, and it can use network sparsity to reduce the amount of calculation, which has a higher potential.

### 5.2. Quantitative analysis of sub-steps acceleration

**Figure 10:**
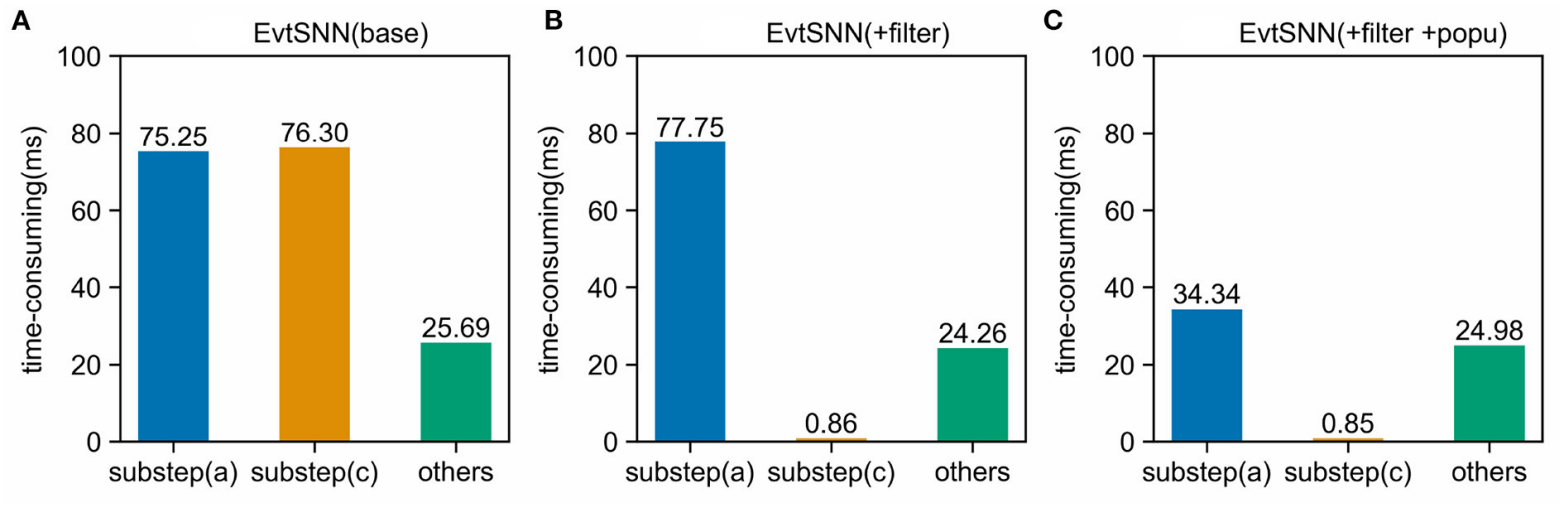
Time-consuming components in simulation. Using Pre-filtering (+filter) avoids unnecessary computation and can significantly reduce the time-consuming of sub-step (c). Enabling population computing (+popu) avoids repeated calculations and speeds up sub-step (a). **(A)** EvtSNN (base), **(B)** EvtSNN (+filter), **(C)** EvtSNN (+filter+popo).

In this section, a dynamic code analysis tool (Clion Profiler) was employed to count the time-consuming of each part. With the 10 kHz sampling frequency and 100 times repeated tasks (same as Section 4.1), the simulation time-consuming composition is shown in Figure 10. Population computing reduces the calculation time of sub-step (a) from 77.75 to 34.34 ms, while pre-filtering reduces the time consumption of sub-step (c) from 76.3 to 0.86 ms. It can be seen that the accelerating effect of pre-filtering is commendable so that the time consumption of sub-step (c) can be neglected. Even poor filtering in extreme cases does not slow down the overall simulation because its computational overhead is negligible.

### 5.3. Acceleration ability in multi-scale network

**Figure 11:**
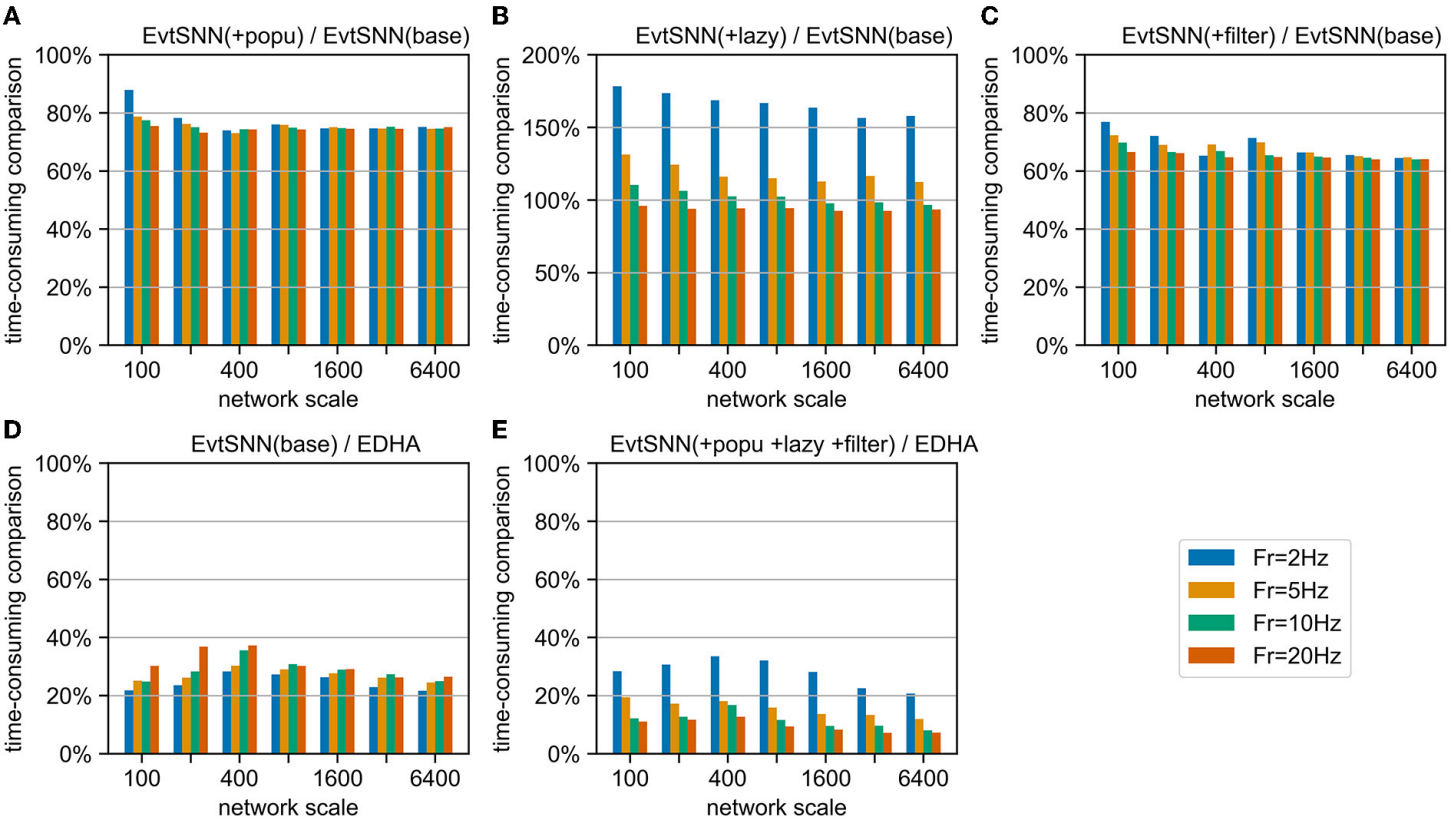
Time-consuming comparison chart. **(A–C)** EvtSNN time-consuming reduction after enabling popu, lazy, and filter, respectively. **(D,E)** Time-consuming comparison of EvtSNN and EDHA with none/all optimizations.

To measure the contribution of each innovation in different network scales, we combined the benchmark and ablation experiment, and the results are shown in Figure 11. Firstly, the acceleration effect of population calculation and pre-filtering under different network scales is relatively stable, reducing the time consumption by about 25 and 35%, respectively compared with EvtSNN (base). Secondly, when the average input frequency (*Fr*) of the neuron group is higher than the output frequency, the delayed update term can have some acceleration effect, otherwise, it will have a negative effect. In addition, after code optimization and rewriting, the speed of EvtSNN (base) is 2.6–4.5 times that of EDHA under the same calculation flow. Finally, EvtSNN using all optimization items is 2.9–14.0 times faster than EDHA.

### 5.4. Limitations

Of course, there are some limitations to our framework. First, as an event-driven framework, EvtSNN has poor model compatibility, requiring the derivation of time-domain equations and the solution of spike firing time. Secondly, the pre-filtering formulation is only used for the neuronal model used. However, pre-filtering formulas for other neuronal models can draw on the derivation process in this paper (3.5).

## 6. Conclusion and future work

Based on the SNN event-driven framework EDHA, a simulator named EvtSNN is introduced. In this paper, two innovations are proposed to speed up the simulation without any accuracy loss. Firstly, repeated calculations are avoided according to the hidden information of the population. Secondly, the unnecessary calculation is filtered by the conditions derived from differential inequality. In the benchmark experiment, without the learning rule, the EvtSNN was the fastest in small network scale simulation (hundreds of neurons). EvtSNN always kept the lead when using the STDP learning rule. In the unsupervised training task of MNIST, EvtSNN only took 56 s to complete one epoch and reached 89.59% accuracy, which is 11.4 times faster than EDHA.

Our work can be further improved. Firstly, large-scale network simulation can be optimized in combination with the clock-driven method. Secondly, multithreading acceleration and parallel computing can be used with the single-layer parallel structure of Inception (Szegedy et al., 2015; Meng et al., 2021) for population-level concurrent acceleration.

## Data availability statement

The original contributions presented in the study are included in the article/supplementary material, further inquiries can be directed to the corresponding author/s.

## Author contributions

LM proposed the idea of EvtSNN. ZT implemented the code of the EvtSNN framework and performed the experiments. Both authors participated in the writing of the manuscript.

## Funding

This work was sponsored by the Blue Project for the University of Jiangsu Province 2021.

## Conflict of interest

The authors declare that the research was conducted in the absence of any commercial or financial relationships that could be construed as a potential conflict of interest.

## Publisher's note

All claims expressed in this article are solely those of the authors and do not necessarily represent those of their affiliated organizations, or those of the publisher, the editors and the reviewers. Any product that may be evaluated in this article, or claim that may be made by its manufacturer, is not guaranteed or endorsed by the publisher.

## References

[B1] Akopyan F. Sawada J. Cassidy A. Alvarez-Icaza R. Arthur J. Merolla P.. (2015). TrueNorth: design and tool flow of a 65 mW 1 million neuron programmable neurosynaptic chip. IEEE Trans. Comput. Aided Design Integr. Circuits Syst. 34, 1537–1557. 10.1109/TCAD.2015.2474396

[B2] Bautembach D. Oikonomidis I. Kyriazis N. Argyros A. (2020). Faster and simpler SNN simulation with work queues, in Proceedings of the International Joint Conference on Neural Networks (Glasgow: Institute of Electrical and Electronics Engineers Inc.). 10.1109/IJCNN48605.2020.9206752

[B3] Davies M. Srinivasa N. Lin T. H. Chinya G. Cao Y. Choday S. H.. (2018). Loihi: a neuromorphic manycore processor with on-chip learning. IEEE Micro 38, 82–99. 10.1109/MM.2018.112130359

[B4] Deng S. Gu S. (2021). Optimal Conversion of Conventional Artificial Neural Networks to Spiking Neural Networks. Available online at: http://arxiv.org/abs/2103.00476

[B5] Diehl P. U. Cook M. (2015). Unsupervised learning of digit recognition using spike-timing-dependent plasticity. Front. Comput. Neurosci. 9:99. 10.3389/fncom.2015.00099 26941637 PMC4522567

[B6] Gerstner W. Kistler W. M. Naud R. Paninski L. (2014). Neuronal Dynamics: From Single Neurons to Networks and Models of Cognition. Cambridge: Cambridge University Press. 10.1017/CBO9781107447615

[B7] Goodman D. Brette R. (2008). Brian: a simulator for spiking neural networks in python. Front. Neuroinformatics 2:5. 10.3389/neuro.11.005.2008 19115011 PMC2605403

[B8] Hazan H. Saunders D. J. Khan H. Patel D. Sanghavi D. T. Siegelmann H. T.. (2018). BindsNET: a machine learning-oriented spiking neural networks library in python. Front. Neuroinformatics 12:89. 10.3389/fninf.2018.00089 30631269 PMC6315182

[B9] LeCun Y. Bottou L. Bengio Y. Haffner P. (1998). Gradient-based learning applied to document recognition. Proc. IEEE 86, 2278–2323. 10.1109/5.726791

[B10] Li S. Zhang Z. Mao R. Xiao J. Chang L. Zhou J. (2021). A fast and energy-efficient SNN processor with adaptive clock/event-driven computation scheme and online learning. IEEE Trans. Circuits Syst. I 68, 1543–1552. 10.1109/TCSI.2021.3052885

[B11] Maass W. (1997). Networks of spiking neurons: the third generation of neural network models. Neural Netw. 10, 1659–1671. 10.1016/S0893-6080(97)00011-7

[B12] Masquelier T. Kheradpisheh S. R. (2018). Optimal localist and distributed coding of spatiotemporal spike patterns through STDP and coincidence detection. Front. Comput. Neurosci. 12:74. 10.3389/fncom.2018.00074 30279653 PMC6153331

[B13] Meng M. Yang X. Bi L. Kim J. Xiao S. Yu Z. (2021). High-parallelism Inception-like spiking neural networks for unsupervised feature learning. Neurocomputing 441, 92–104. 10.1016/j.neucom.2021.02.027

[B14] Mo L. Chen X. Wang G. (2021). Edha: Event-driven high accurate simulator for spike neural networks. Electronics 10:2281. 10.3390/electronics10182281

[B15] Moradi S. Qiao N. Stefanini F. Indiveri G. (2018). A scalable multicore architecture with heterogeneous memory structures for dynamic neuromorphic asynchronous processors (DYNAPs). IEEE Trans. Biomed. Circuits Syst. 12, 106–122. 10.1109/TBCAS.2017.2759700 29377800

[B16] Neftci E. O. Mostafa H. Zenke F. (2019). Surrogate gradient learning in spiking neural networks: bringing the power of gradient-based optimization to spiking neural networks. IEEE Signal Process. Mag. 36, 51–63. 10.1109/MSP.2019.2931595

[B17] Paszke A. Gross S. Massa F. Lerer A. Bradbury J. Chanan G.. (2019). PyTorch: an imperative style, high-performance deep learning library, in Advances in Neural Information Processing Systems, Vol. 32 (Vancouver, BC).

[B18] Pei J. Deng L. Song S. Zhao M. Zhang Y. Wu S.. (2019). Towards artificial general intelligence with hybrid Tianjic chip architecture. Nature 572, 106–111. 10.1038/s41586-019-1424-8 31367028

[B19] Pham Q. T. Nguyen T. Q. Hoang P. C. Dang Q. H. Nguyen D. M. Nguyen H. H. (2021). A review of SNN implementation on FPGA, in 2021 International Conferenceon Multimedia Analysis and Pattern Recognition, MAPR 2021 - *Proceedings* (Hanoi: Institute of Electrical and Electronics Engineers Inc.). 10.1109/MAPR53640.2021.958524527295638

[B20] Sengupta A. Ye Y. Wang R. Liu C. Roy K. (2019). Going deeper in spiking neural networks: VGG and residual architectures. Front. Neurosci. 13:95. 10.3389/fnins.2019.00095 30899212 PMC6416793

[B21] Shen J. Ma D. Gu Z. Zhang M. Zhu X. Xu X.. (2016). Darwin: a neuromorphic hardware co-processor based on Spiking Neural Networks. Sci. China Inform. Sci. 59, 1–5. 10.1007/s11432-015-5511-7

[B22] Stimberg M. Brette R. Goodman D. F. (2019). Brian 2, an intuitive and efficient neural simulator. eLife 8:e47314. 10.7554/eLife.47314.028 31429824 PMC6786860

[B23] Szegedy C. Liu W. Jia Y. Sermanet P. Reed S. Anguelov D.. (2015). Going deeper with convolutions, in Proceedings of the IEEE Computer Society Conference on Computer Vision and Pattern Recognition (Boston, MA: IEEE Computer Society), 1–9. 10.1109/CVPR.2015.7298594

